# The SGLT-2 inhibitor empagliflozin improves myocardial strain, reduces cardiac fibrosis and pro-inflammatory cytokines in non-diabetic mice treated with doxorubicin

**DOI:** 10.1186/s12933-021-01346-y

**Published:** 2021-07-23

**Authors:** Vincenzo Quagliariello, Michelino De Laurentiis, Domenica Rea, Antonio Barbieri, Maria Gaia Monti, Andreina Carbone, Andrea Paccone, Lucia Altucci, Mariarosaria Conte, Maria Laura Canale, Gerardo Botti, Nicola Maurea

**Affiliations:** 1 Division of Cardiology, Istituto Nazionale Tumori-IRCCS-Fondazione G. Pascale, Naples, Italy; 2 Breast Unit, Istituto Nazionale Tumori-IRCCS-Fondazione G. Pascale, Naples, Italy; 3 SSD Sperimentazione Animale, Istituto Nazionale Tumori-IRCCS-Fondazione G. Pascale, Naples, Italy; 4 Department of Translational Medical Sciences, University of Naples “Federico II”, Naples, Italy; 5 Department of Precision Medicine, University of Campania ‘Luigi Vanvitelli’, Via L. De Crecchio 7, 80138 Naples, Italy; 6 Cardiology Division, Azienda USL Toscana Nord-Ovest, Versilia Hospital, Lido Di Camaiore, Italy; 7 Scientific Direction, Istituto Nazionale Tumori-IRCCS-Fondazione G. Pascale, Naples, Italy

**Keywords:** EMPA, Cardio-Oncology, Doxorubicin, Inflammation, Interleukins

## Abstract

**Background:**

Empagliflozin (EMPA), a selective inhibitor of the sodium glucose co-transporter 2, reduced the risk of hospitalization for heart failure and cardiovascular death in type 2 diabetic patients in the EMPA‐REG OUTCOME trial. Recent trials evidenced several cardio-renal benefits of EMPA in non-diabetic patients through the involvement of biochemical pathways that are still to be deeply analysed. We aimed to evaluate the effects of EMPA on myocardial strain of non-diabetic mice treated with doxorubicin (DOXO) through the analysis of NLRP3 inflammasome and MyD88-related pathways resulting in anti-apoptotic and anti-fibrotic effects.

**Methods:**

Preliminary cellular studies were performed on mouse cardiomyocytes (HL-1 cell line) exposed to doxorubicin alone or combined to EMPA. The following analysis were performed: determination of cell viability (through a modified MTT assay), study of intracellular ROS production, lipid peroxidation (quantifying intracellular malondialdehyde and 4-hydroxynonenal), intracellular Ca^2+^ homeostasis. Moreover, pro-inflammatory studies were also performed: expression of NLRP3 inflammasome, MyD88 myddosome and p65/NF-κB associated to secretion of cytokines involved in cardiotoxicity (Interleukins 1β, 8, 6). C57Bl/6 mice were untreated (Sham, n = 6) or treated for 10 days with doxorubicin (DOXO, n = 6), EMPA (EMPA, n = 6) or doxorubicin combined to EMPA (DOXO-EMPA, n = 6). DOXO was injected intraperitoneally. Ferroptosis and xanthine oxidase were studied before and after treatments. Cardiac function studies, including EF, FS and radial/longitudinal strain were analysed through transthoracic echocardiography (Vevo 2100). Cardiac fibrosis and apoptosis were histologically studied through Picrosirius red and TUNEL assay, respectively and quantified through pro-collagen-1α1, MMP-9 and Caspase-3 expression. Tissue NLRP3, MyD88 and cytokines were also quantified before and after treatments through ELISA methods.

**Results:**

Cardiomyocytes exposed to doxorubicin increased the intracellular Ca^2+^ content and expression of several pro-inflammatory markers associated to cell death; co-incubation with EMPA reduced significantly the magnitude of the effects. In preclinical study, EMPA increased EF and FS compared to DOXO groups (p < 0.05), prevented the reduction of radial and longitudinal strain after 10 days of treatment with doxorubicin (RS) 30.3% in EMPA-DOXO vs 15.7% in DOXO mice; LS − 17% in EMPA-DOXO vs – 11.7% in DOXO mice (p < 0.001 for both). Significant reductions in ferroptosis, xanthine oxidase expression, cardiac fibrosis and apoptosis in EMPA associated to DOXO were also seen. A reduced expression of pro-inflammatory cytokines, NLRP3, MyD88 and NF-kB in heart, liver and kidneys was also seen in DOXO-EMPA group compared to DOXO (p < 0.001).

**Conclusion:**

EMPA reduced ferroptosis, fibrosis, apoptosis and inflammation in doxorubicin-treated mice through the involvement of NLRP3 and MyD88-related pathways, resulting in significant improvements in cardiac functions. These findings provides the proof of concept for translational studies designed to reduce adverse cardiovascular outcomes in non-diabetic cancer patients treated with doxorubicin.

## Background

Doxorubicin (DOXO) induced-cardiotoxicity is a well-known adverse event in cancer patients [1, 2]. As recently described in literature, DOXO-induced cardiotoxicity involves pro-inflammatory interleukins, pro-oxidative markers, ferroptosis, topoisomerase IIβ inhibition and mitochondrial dysfunction [3–5]. Improvement in knowledge of DOXO-induced pathophysiology pushes the search for new potential cardioprotective agents able to prevent cardiotoxic events.

EMPA (EMPA) (Jardiance, Boehringer Ingelheim) is a sodium-glucose cotransporter 2 (SGLT-2) inhibitor with hypoglycaemic and anti-oxidant effects [6]. The beneficial properties of EMPA involves both indirect and direct effects in heart tissue, such as the reduction of 8-iso prostaglandin f2α (a product of lipid peroxidation), blunted the activation of mitogenic stress pathways such ERKs, JNKs, and p38 MAPKs [7].

In EMPA-REG Outcome trial, which enrolled about 7000 patients affected by type 2 diabetes, deaths from cardiovascular causes were 3.7% in patients treated with EMPA versus 5.9% in patients receiving placebo, which corresponds to a relative risk reduction of cardiovascular diseases of 38% [8–10]. Interestingly, EMPA exerts beneficial effects also in non-diabetic patients, as described in recent clinical trials named EMPEROR-Reduced/preserved [11].

Given the crucial role of the heart pro-inflammatory microenvironment in the genesis of DOXO-related cardiotoxicity, we evaluated the impact of EMPA on DOXO-induced cardiotoxicity and studied the putative beneficial effects on fibrosis, cardiac functions, and expression of pro-inflammatory cytokines both in cardiomyocytes and mouse models.

## Methods

### Cell viability

Firstly, we aimed to evaluate the effects of EMPA combined to DOXO in cell cultures of cardiomyocytes (HL-1 adult mouse cells derived from American Type Culture Collection, Manassas, VA, USA), estrogen-responsive and triple-negative breast cancer cells (MCF-7 and MDA-MB-231 cell lines, derived from American Type Culture Collection, Manassas, VA, USA). To evaluate the cytotoxic or cytoprotective effects of EMPA, the mitochondrial dehydrogenase activity was quantified through a modified MTT [3-(4,5-dimethyldiazol-2-yl)-2,5-diphenyl tetrazolium bromide] method, called MTS assay, according to the manufacturer’s instructions (Dojindo Molecular Technologies Inc., Rockville, MD, USA) [12]. Briefly, HL-1 cells were grown in a complete medium constituted by Claycomb medium, 10% V/v heat-inactivated foetal bovine serum, 2 mM l-glutamine, 100 U/ml penicillin and 100 μg/ml streptomycin in 96-well plates (density of 10,000 cells/well) at 37 °C in a humidified 5% CO_2_ atmosphere. MCF-7 human breast cancer cells (ERα + , PR + , HER2-) were cultured in Dulbecco’s modified Eagle’s medium (DMEM) supplemented with 10% fetal bovine serum (FBS), 2 mM glutamine, 100 units/mL penicillin and 100 units/mL streptomycin. Triple negative MDA-MB-231 (ATCC® HTB-26™) cells were grown in ATCC-formulated Leibovitz’s L-15 Medium supplemented with 10% fetal bovine serum (FBS) (HyClone™, GE Healthcare Life Sciences, Milan, Italy) and Penic illin-Streptomycin (100 U/mL, Gibco®, Milan, Italy). Cell cultures were maintained in a humidified atmosphere of 95% air and 5% CO2 at 37 °C.

After 24 h of appropriate growth, cells were exposed to: DOXO (0.1 to 50 µM); EMPA (50, 100 and 500 nM); DOXO-EMPA (both drugs combined). Cells were than incubated for 24 h with each drug under standard growth conditions. Cells were then washed three times with phosphate buffered solution (PBS) at pH 7.4 and then incubated with 100 μl of an MTT solution (0.5 mg/ml in cell culture medium) for 4 h at 37 °C. Absorbance readings were acquired at a wavelength of 450 nm with the Tecan Infinite M200 plate-reader (Tecan Life Sciences Home, Männedorf, Switzerland) using I-control software (Tecan). Relative cell viability (%) was calculated with the following formula [A]test/[A]control × 100, where “[A]test” is the absorbance of the test sample, and “[A]control” is the absorbance of the control cells incubated solely in culture medium.

### Quantification of intracellular reactive oxygen species (iROS)

Quantification of iROS was performed by using a conventional fluorescent probe (DCFH-DA) as described elsewhere [13]. Cardiomyocytes (5 × 10^3^ cells/well) were seeded in a 24-well plate and allowed to grow for 24 h; after, cells were pre-treated or not with EMPA at 10, 50 and 500 nM for 4 h. Cardiomyocytes pre-treated with gallic acid (50 µM) served as positive control. After, cells were incubated with 5 μM DCFH-DA in PBS for 30 min. Then, DCFH-DA was removed from each well and cells were stimulated with 40 ng/mL of lipopolysaccharides (LPS, as internal control) or DOXO at 100 nM for 12 h. Cell fluorescence was measured using a microplate spectrofluorometer. Intracellular antioxidant activity was expressed as percentage of control cells. The concentration of DOXO used were within the range of 25–250 nM, calculated for steady-state plasma concentrations of DOXO, intravenously administered, in cancer patients at the common therapeutic dosage of 15–90 mg/m^2^ [14, 15].

### Lipid peroxidation

To study the putative anti-oxidant effects of EMPA, HL-1 cells were grown as described above. Subsequently, 5 × 10^3^ cells/well were seeded in a 24-well plate and allowed to grow for 24 h and exposed to DOXO (100 nM) or LPS (40 ng/ml, as positive control in inflammation in human cells, as described by our group [12, 13]) for 6 h or pre-treated for 4 h with EMPA (10, 50 and 500 nM) or with gallic acid (50 µM) as anti-oxidant positive control. After centrifugation at 800 × g for 5 min, the supernatant was evaluated for malondialdehyde (MDA) and 4-hydroxy 2-hexenal (4-HNA) using commercial kits with a spectrophotometer according to the manufacturer's protocols (Sigma Aldrich, Milan, Italy).

### Nitric oxide assay

To evaluate the effects of EMPA on the release of nitric oxide from HL-1 cells, we analysed the release of nitrite, which is a stable product of nitric oxide in aqueous medium, using the Griess Reagent System (Promega, Madison, WI, USA) as described elsewhere [16]. Cells were treated as described before; after treatment, the culture medium was then mixed with an equal volume of sulfanylamide solution (1% v/v in 5% v/v phosphoric acid) and of N-1-naphtylethylenediamine dihydrochloride solution (0.1% v/v in water). Absorbance was measured at 540 nm with a spectrophotometer.

### Intracellular Ca^2+^ assay

Cardiomyocytes exposed to DOXO increases the intracellular calcium concentration due overproduction of iROS [4]. We quantified the intracellular Ca^2+^ in HL-1 cells by using the fluorescence dye Fluo-3 AM, according to the manufacturer's protocol. Cardiomyocytes were untreated or treated as described before. After incubation, cells were loaded with 5 µM Fluo-3 AM at 37 °C for 30 min in the dark, and then washed three times with PBS (pH 7.4) to remove the excess dye. Fluo-3 chelated with calcium produces fluorescence that was quantified with a spectrofluorometer at excitation and emission wavelengths of 488 nm and 525 nm, respectively.

### Anti-inflammatory studies

#### Cytokine assay

The expression of IL-6, IL-8 and IL-1β was performed through ELISA method, as described elsewhere [12]. Briefly, HL-1 cells were grown as described above. After incubation for 24 h and starvation in serum-free medium for 2.5 h, HL-1 cells were treated or not with EMPA in doses ranging from 10 to 500 nM for 4 h before exposure to LPS (40 ng/ml) or DOXO (100 nM) for 12 h to stimulate inflammation. After exposure, supernatants were collected, centrifuged to pellet any detached cells and measured using IL-1β, IL-6 and IL-8 ELISA kits according to the manufacturer’s instructions (Sigma Aldrich, Milan, Italy).

#### Leukotriene B4 assay

To quantify leukotrienes B4 (LTB4), cardiomyocytes were treated as described before; after treatments, cells were incubated at 37 °C for 30 min with serum-free medium containing a solution constituted by 5 μM of the calcium ionophore A23187, 1.6 mM of CaCl2 and 10 μM of arachidonic acid. Arachidonate was used as precursor of leukotriene synthesis. Immuno reactive LTB4 was quantified with an ELISA procedure (Cayman Chemical) according to the supplier's instructions [17].

#### p65/NF-kB expression

Cardiomyocytes were treated with DOXO (100 nM) or EMPA (10, 50 and 500 nM) or DOXO and EMPA for 24 h. After, nuclear extracts were analysed using the TransAM p65/NF-κB transcription factor assay kit (Active Motif, Carlsbad, CA, USA), according to the manufacturer’s recommendations [18]. Data were expressed as the percentage of p65/NF-kB DNA binding versus control (untreated) cells.

#### NLRP3 and MyD88 expression

Cardiomyocytes were treated with DOXO (100 nM) or EMPA (10, 50 and 500 nM) or DOXO and EMPA for 24 h. After treatments, cells were harvested and lysed in complete lyses buffer (50 mM Tris–HCl, pH 7.4, 1 mM EDTA, 100 mM NaCl, 20 mM NaF, 3 mM Na3 VO4, 1 mM PMSF, and protease inhibitor cocktail). After centrifugation, supernatants were collected and treated to the quantification of MyD88 (Mouse MyD88 ELISA Kit, Abcam, Italy) and NLRP3 (Mouse NLRP3 ELISA Kit, Aviva Systems Biology). For mouse MyD88 ELISA the sensitivity was < 10 pg/ml and range of detection was 156–10,000 pg/ml; for mouse NLRP3 ELISA assay, the sensitivity was < 0.078 ng/mL and range of detection was 0.156–10 ng/mL.

#### Confocal laser scanning microscope (CLSM) imaging

HL-1 cells were cultured as described above. After, 5 × 10^3^ cells/well were seeded in a 24-well plate and allowed to grow for 24 h and untreated (control) or treated with DOXO (100 nM) or EMPA (100 nM) or DOXO (100 nM) combined with EMPA (100 nM) for one day. Then, cells were thoroughly rinsed three times with PBS and fixed with 2.5% glutaraldehyde in PBS for 20 min, as described elsewhere [19]. After washing three times with PBS, cells were permeabilized with 0.1% Triton-X100 in PBS for 10 min and then washed three times with PBS. Subsequently, cells were blocked with 1% BSA in PBS for 20 min. After three washes with PBS, cells were incubated with a Rabbit polyclonal antibody against p65/NF-kB (clone ab16502 Abcam) diluted 1:200 in 1% BSA for 1 h. After washing, cells were incubated for 1 h with Goat Anti-Rabbit secondary antibody IgG H&L (FITC) (clone ab6717, AbCam) diluted 1:1000 in 1% BSA. Nuclear staining was obtained through the use of NUCLEAR-ID® Red DNA stain (Enzo Life Technology, Milan, Italy) diluted 1:2000 in PBS for 15–30 min at 37 °C. After washing in PBS, cells were blocked with 1% BSA in PBS for 20 min. A confocal microscope (C1 Nikon) equipped with EZ-C1 software for data acquisition was used (60 × oil immersion objective). Expression of p65/NF-kB and nucleus were imaged through excitation/emission at 492/518 nm and 566/650 nm, respectively.

### Animal models

Twenty-four female C57Bl/6 mice (6 weeks/age) were purchased from Harlan, San Pietro al Natisone (Italy). Mice were housed 6 per cage and maintained on a 12-h light to 12 h dark cycle (lights on at 7.00 am) in a temperature-controlled room (22 ± 2 °C) and with food and water ad libitum. Preclinical experimental protocols were in accordance with EU Directive 2010/63/EU for animal experiments, and Italian D.L.vo 26/2014 low; were approved by Ministry of Health with authorization number 1467/17-PR of the 13–02-2017, and institutional ethics committees: Organismo preposto al benessere degli animali (OPBA). After 1 week of growth, mice were randomized for weight-adjusted treatment. Mice were divided in 4 experimental groups (n = 6/group) (i) 100 μl saline solution (Sham); (ii) DOXO at 2.17 mg/kg/day through intraperitoneal administration (i.p); (iii) EMPA 10 mg/kg/day through oral gavage; (iv) EMPA/DOXO in combination (at the same concentration of each drug tested alone). Treatments were performed according to our protocol recently published were we evaluated the cardioprotective effects of Ranolazine against cardiotoxicity of doxorubicin for 10 days [20]; in fact, also in this case, in group of combinatorial treatment EMPA/DOXO, mice were treated with EMPA alone for 3 days and the remaining 7 days also in combination to DOXO. Another work investigated on the initial damages of doxorubicin and trastuzumab administration at low doses in mice with significant changes in cardiac apoptosis, necrosis and fibrosis leading to reduced cardiac functions after 7 days of treatment [21]. Notably, used short-term treatment of doxorubicin is more than sufficient to evaluate myocardial dysfunction in mice; in fact, in the same experimental procedure showed by Tocchetti G et al. [20], and Fedele et al. [22] in C57BL6 mice, doxorubicin treatment for 7 days produced left ventricular dilation and decreased echo-measured fractional shortening (FS) as well as detectable apoptosis and inflammation in myocardial tissues. Blood glucose determination were performed via puncture of the tail vein before and after treatments by a glucometer (Model NC).

#### Transthoracic echocardiography

To assess cardiac function in vivo we performed non-invasive transthoracic echocardiography in sedated mice using a Vevo 2100 high-resolution imaging system (40-MHz transducer; Visualsonics, Toronto, ON, Canada) as described in literature [21, 23]. Mice were anaesthetized with tiletamine (0.09 mg/g), zolazepam (0.09 mg/g), and 0.01% atropine (0.04 mL/g). After, animals were sedated and placed in supine position on a temperature-controller surgical table to maintain rectal temperature at 37 °C, continual ECG monitoring was obtained via limb electrodes. Cardiac function was evaluated at basal conditions and at 2 and 10 days of treatments. Left ventricular echocardiography was assessed in parasternal long-axis views at a frame rate of 233 Hz. Notably, we measured the strain in parasternal views because the apical view is difficult to perform in small animal [24]; this method was in line with other studies for STE analyses that were performed on parasternal long-axis B-mode loops using a VisualSonics Vevo 2100 system (VisualSonics) [25–27]. Image depth, width, and gain settings were optimized to improve image quality. End-systole and end-diastole dimensions were defined as the phases corresponding to the ECG T wave, and to the R wave, respectively. M-mode LV internal dimensions, diastolic (LVID,d) and LV internal dimensions, systolic (LVID,s) were averaged from 3 to 5 beats. LVID,d and LVID,s were measured from the LV M-mode at the mid papillary muscle level. Fractional shortening percentage (% FS) was calculated as [(LVID, d-LVID, s)/LVID, d] X 100, and ejection fraction percentage (% EF) was calculated as [(EDvol-ESvol)/EDvol] X100. The strain was expressed as percentage. The analysis start with acquired B-mode loops and were imported into the Vevo Strain software. Three consecutive cardiac cycles were selected and the endocardium traced. Upon adequate tracing of the endocardium, an epicardial trace was added. ST based strain allowed assessment of strains specific to 6 myocardial segments per LV view. Internally, 10 or plus points were measured for each of the 6 segments, resulting in 48 data points in total. Strain and SR are useful in the detection of regional myocardial function. The strain is evaluated on long-axis views as well as: radial and longitudinal. Radial strain (RS), defined as the percent change in myocardial wall thickness is a positive curve reflecting increasing myocardial thickness during systole and diminishing wall thickness during diastole and represent myocardial deformation toward the centre of the LV cavity. Longitudinal strain (LS) detects the percent change in length of the ventricle, typically measured from the endocardial wall in the long-axis view. Myocardial deformation rate, expressed in 1/s, was also calculated. Notably, we measure LV, diastolic and systolic volumes in the one-dimensional view following the proper instructions of “Small Animal Echocardiography using the Vevo® 2100 Imaging System” [28] and also in agree with our previous similar work [20].

#### Isolation of cytosolic and mitochondrial fractions in heart tissue

Isolation of cytosolic and mitochondrial fractions was performed using heart tissues wherein the Mitochondria/Cytosol Fractionation Kit (ab65320, Abcam) was used for cytosol fractionation and the Mitochondria Isolation Kit (P507L, 101 Bio) for mitochondrial fractionation in accordance with the manufacturer’s instructions.

#### Ferroptosis assay: measurements of mitochondrial lipid peroxidation using MitoPeDPP and MDA content in cytosolic and mitochondrial fractions

Increasing evidence indicates that ferroptosis plays a critical role in doxorubicin-induced cardiotoxicity [29–31]; therefore, we aimed to test the effect of EMPA on ferroptosis during DOXO treatment. Mitochondrial lipid peroxidation was measured using MitoPeDPP test (a fluorescence probe that specifically reacts with lipid peroxides in the mitochondrial inner membrane (Dojindo). Briefly, mitochondrial fraction was incubated for 30 min at 37 °C in a solution at 0.5 μM MitoPeDPP. After incubation, lipid peroxides in the mitochondrial inner membrane were fluorometrically measured (excitation 452 nm and emission at 470 nm) using a Tecan Infinite M200 plate-reader (Tecan Life Sciences Home, Männedorf, Switzerland) equipped with a I-control software (Tecan). MDA levels in cytosolic and mitochondrial fraction of heart tissues were measured using commercial kit (MDA ELISA kit; Sigma Aldrich; Milan, Italy) were lipid peroxidation is determined by the reaction of MDA with thiobarbituric acid (TBA) to form a colorimetric (532 nm)/fluorometric (λex = 532/λem = 553 nm) product, proportional to the MDA content.

#### Xanthine oxidase activities

The recent study by Tanaka et al. showed that the increased oxidative stress induced by tissue xanthine oxidase activation is deeply involved in doxorubicin-induced cardiotoxicity [31]. The xanthine oxidase activities in heart tissues were measured using the horseradish peroxidase-linked Amplex Red fluorescence assay kit (Molecular Probes, Invitrogen Detection Technologies), according to the manufacturer's instructions. In brief, heart tissues were homogenized in T-PER Tissue Protein Extraction (Thermo Fisher Scientific, USA) and centrifuged at 14,000 g for 10 min at 4 °C. Supernatant was added to a working solution composed by Amplex Red reagent (100 μM), xanthine (0.2 mM), and horseradish peroxidase type (0.4 U/ml), incubated at 37 °C for 30 min; produced H_2_O_2_ was measured. Fluorescence readings were made in triplicate in a 96-well plate at Ex/Em = 540/590 nm. The XO activity was corrected by the protein concentration of the supernatant measured by a Bradford assay.

#### Anti-inflammatory studies in tissue extracts

After treatments, heart, liver and left kidney were weighed and treated for quantification of cytokines, MyD88 and NLRP3. The heart tissues were cut in transverse section into two parts. The basal parts of the hearts, and the whole liver and left kidney were snap-frozen in dry ice until tissue homogenization, which was carried out in 0.1 M PBS (pH 7.4) containing 1% Triton X-100, protease inhibitor cocktail and processed using a high intensity ultrasonic liquid processor. The homogenates were centrifuged at 4 °C and supernatants were used to determine tissue markers. The apical parts of the heart sections were fixed in 10% neutral buffered formalin for 48 h for the cardiac fibrosis and apoptosis assays Methods for quantification in interleukins in tissue extracts were followed using the appropriate ELISA kits for mouse IL-1β, IL-8 and IL-6 detections, as used in the cellular experiments, according to the manufacturer’s instructions; results are expressed as pg of interleukin/mg of tissue. The MyD88 and NLRP3 expression in heart tissues were performed through the use of the same kits described for cellular experiments and results are expressed as pg of MyD88 or pg of NLRP3/mg of protein.

#### Cardiac fibrosis: collagen type I expression in cardiac tissue

For ex vivo analyses, hearts were excised and fixed in 10% neutral buffered solution. The myocardial tissue was formalin-fixed and paraffin-embedded for morphometry and immunohistochemistry. General morphology was studied using haematoxylin–eosin staining. To measure collagen content, we deparaffinised 6 µm-thick cross sections and stained them with Picrosirius red (Carlo Erba Laboratories, Milan, Italy). The collagen volume fraction was expressed as the mean percentage of Picrosirius red-stained tissue areas divided by total tissue area in the same field and was evaluated in 15 fields at 60× magnification. The positively stained (red) fibrotic area was measured with a computer-assisted image analysis system (Nikon NIS Elements. Nikon Instruments, Melville, NY, USA). To measure capillary density, we incubated sections overnight with biotinylated Bandeira easimplicifolia Isolectin-I (Sigma-Aldrich Co., St Louis, MO, USA) followed by tyramide signal amplification enhancement (PerkinElmer Inc., Waltham, MA, USA). Capillaries were visualized by 3,3′-diaminobenzidine tetrahydrochloride, counted and expressed as the number of capillaries per mm^2^. In order to quantify the total collagen content in the heart tissues, measurement of pro-collagen 1α1 (an established biomarker of cardiac fibrosis) was performed using the Mouse Pro-Collagen I alpha 1 CatchPoint SimpleStep ELISA Kit from Abcam (ab229425). Tissues were homogenized, after protein quantification (Bradford assay) 100 µg of proteins were assayed according to manufacturer’s instruction. Fluorescence were measured at 530/590 using a 96-well plate reader Tecan Infinite M200 plate-reader (Tecan Life Sciences Home, Männedorf, Switzerland).

#### Cardiac apoptosis through TUNEL and caspase-3 assay and MMP-9 expression

Cardiac sections measuring 6 μm were examined for the presence of apoptotic cardiomyocytes by TdT-mediated dUTP nick-end labelling (TUNEL) assay using a Promega Dead End™ colorimetric TUNEL system (Promega, Madison, WI, USA) with a streptavidin-peroxidase system. Controls were obtained by omitting the TdT enzyme from the reaction mixture. The percentage of TUNEL-positive myocytes was determined by counting 10 random fields per section under a microscope (Nikon NIS Elements). Using this procedure, apoptotic nuclei were stained dark brown. Labelled nuclei were counted and expressed as the percentage of positively stained cells. The Caspase-3 activity in heart tissue was measured using a Caspase-3 colorimetric assay kit (Clontech, USA) according to the manufacturer’s instructions (results were reported as ng of Caspase-3/g of tissue). MMP-9 were associated with collagen maturation in heart failure, demonstrating the important role of these enzymes in fibrosis through collagen configuration, activation, and deposition. Therefore, we quantified MMP-9 in heart tissues through Mouse MMP9 ELISA Kit (ab253227, AbCam, Milan, Italy) following the manufacturer’s instructions; results are expressed as pg of MMP-9/mg of protein (determined through Bradford assay).

#### SGLT-2 expression through western blot analysis

Same studies indicated that human and murine cardiomyocytes express SGLT-2 [32], although other studies indicate its absence [33]. To confirm this data, we analysed SGLT-2 expression in heart and renal (as positive control) tissues of mice. For preparation of Protein Lysates from Mouse Tissues and western blot analysis, frozen mouse kidney and heart tissues (50 mg) were cut on dry ice and resuspended in 1 mL of RIPA buffer (Tris–HCl pH 8.0, 50 mM sodium chloride, 1.0% NP-40, 0.5% sodium deoxycholate, 0.1% sodium dodecyl sulfate, protease inhibitors). Tissues were homogenized and transferred to a new tube. Later they were centrifuged at 13,000 rpm at 4 °C for 20 min and the supernatants were collected. The protein concentrations were determined by colorimetric assay (Biorad, Italy). The tissues extracts were diluted 1:1 in sample buffer 2X Laemli (0.217 M Tris–HCl pH 8.0, SDS 52.17%, 17.4% glycerol, 0,026% bromophenol blue, 8.7% β-mercaptoethanol), and then were boiled for 3 min. Equal amounts of protein (50 µg) were run and separated by SDS-PAGE gel. Primary antibody used was: SGLT-2 (Thermo Fisher Scientific), while GAPDH (Cell Signaling) antibody was used as loading control.

### RNA isolation and RT-PCR

Mice heart and kidney tissues (50 mg each) were submerged in RNA stabilizing solution (RNAlater®). Samples were then minced and RNA were isolated using TRIzol Reagent (Invitrogen-Life Technologies) according to the manufacturer's protocol and subsequently reverse transcribed through Super Script VILO kit (Invitrogen). Relative mRNA levels were performed by qRT-PCR using iQ SYBR® Green Supermix (Bio-Rad Laboratories). Specific mouse primers used for qRT-PCR were: SGLT-2 Fw: 5′- TAGTTGGAAGGCTCTGGGTG-3′; SGLT-2 Rev: 5′- CCCAACTAGTCCCCAGAAGG-3′. Data were normalized with GAPDH Fw: 5′- CCCCAATGTGTCCGTCGTG-3′; GAPDH Rev: 5′- GCCTGCTTCACCACCTTCT-3′ by using ∆∆CT method. Results were expressed as fold-change compared to the control.

### Statistical analysis

Continuous data were expressed as mean ± SD. Nonparametric tests were used both for paired and unpaired comparisons. Repeated measures ANOVA was used for all baseline to end-of-study comparisons. A p value < 0.05 was considered significant.

## Results

### Cell viability and calcium homeostasis

**Fig. 1:**
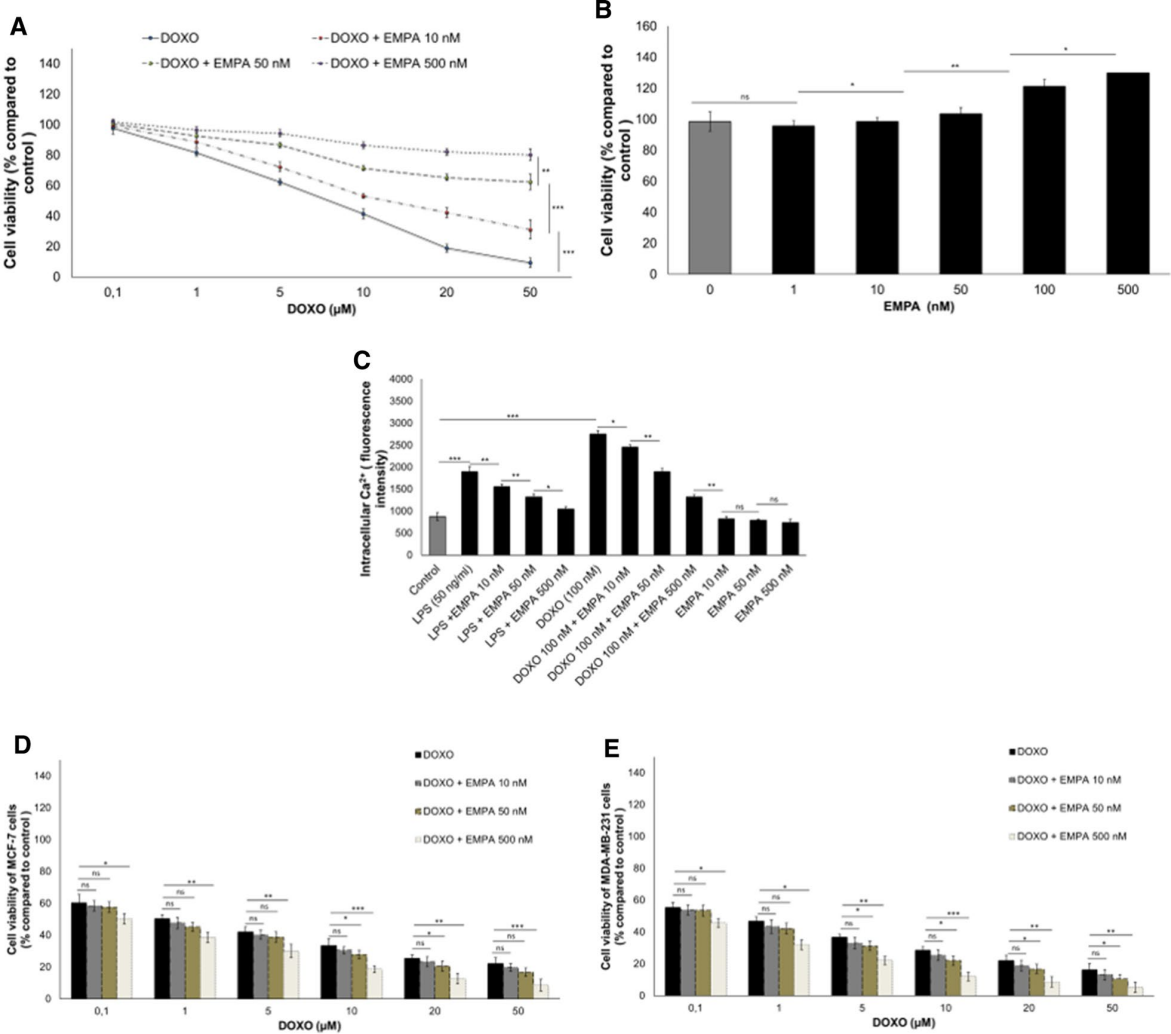
EMPA exerts a cytoprotective effect on cardiomyocytes exposed to doxorubicin (DOXO) through the reduction of intracellular Ca2^+^ overload in a concentration-dependent manner. 10 × 10^3^ HL-1 cells/well were seeded in 96-well plate and were cultured under different conditions. **A** cell viability of cardiomyocytes treated with DOXO (from 0.1 to 50 µM), alone or combined with EMPA (EMPA) (10, 50 or 500 nM) (n = 6 for each concentration. One-way ANOVA analysis). **B** Cardiomyocyte viability under exposure to EMPA from 1 to 500 nM. (n = 6 for each concentration. One-way ANOVA analysis). **C** For quantification of intracellular calcium (Ca2^+^), 5 × 10^3^ cells/well were seeded in a 24-well plate and allowed to grow for 24 h; after, cardiomyocytes were not exposed (control) or exposed to EMPA (10, 50, 500 nM), lipopolysaccharides (LPS) (50 ng/ml) or DOXO (100 nM) alone or with EMPA at 10, 50 and 500 nM. (n = 3 for each group). One-way ANOVA analysis). Values are expressed ± SD. ***P < 0.001; **P < 0.01; *P < 0.05; ns: not significant

Incubation with DOXO decreased the viability of cardiomyocytes in a concentration-dependent manner, with an IC_50_ below 10 µM, whereas DOXO and EMPA co-incubation increased cell viability (Fig. 1A). For example, the viability of cardiomyocytes exposed to DOXO at 50 µM plus EMPA at 10, 50 or 500 nM was 21.8%, 53.1% and 71%, respectively, higher than that of cardiomyocytes exposed to DOXO alone. In addition, cell viability was 18.8% higher in cardiomyocytes treated with EMPA alone at 500 nM than in control cells (Fig. 1B). Moreover, [Ca^2+^]i (intracellular calcium) production was significantly higher in cardiomyocytes treated with DOXO and lipopolysaccharides (LPS) than in untreated cells (p < 0.001 for both) (Fig. 1C). At 24 h of incubation, the mean [Ca^2+^]i of cardiomyocytes incubated with DOXO and EMPA at 10, 50 and 500 nM, was about 8%, 40% and 53% lower than the mean [Ca^2+^]i in cells treated with DOXO alone (P < 0.001) (Fig. 1C). The same results were obtained under pro-inflammatory conditions in LPS- and LPS/EMPA-treated cells (Fig. 1C). As control, we evaluated the effects of EMPA on the anticancer efficacy of DOXO in human estrogen-responsive (Fig. 1D) and triple negative breast cancer cells (Fig. 1E); in line with literature [34], EMPA did not affects the anticancer effects of DOXO in breast cancer cells, indeed, at some concentrations it seems to increase slightly the cytotoxicity of DOXO. These preliminary results, although already confirmed by recent research [34], are of great interest and will be the performed by our group in further in-depth studies.

### Intracellular reactive oxygen species (iROS), nitric oxide and lipid peroxidation

**Fig. 2:**
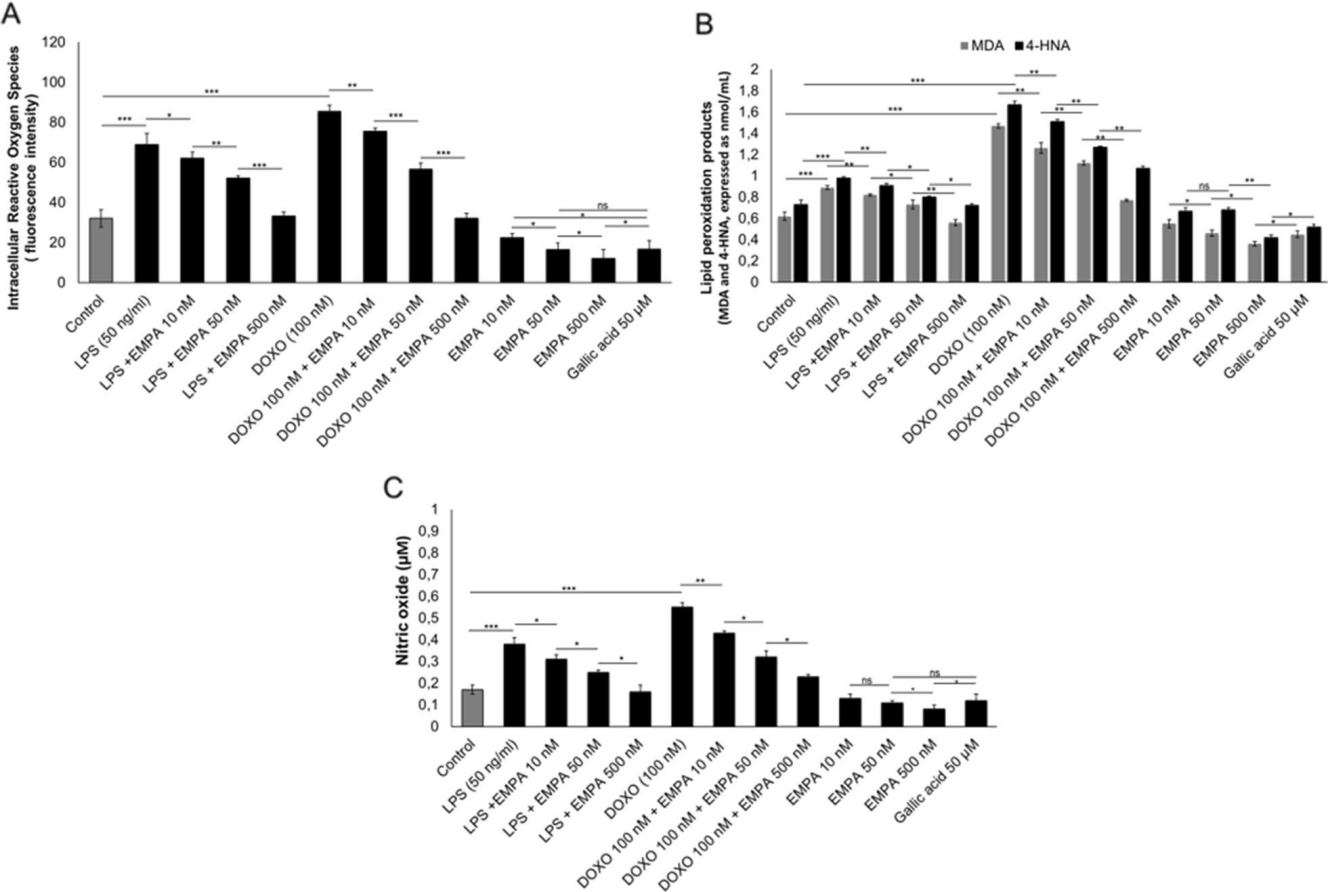
EMPA has anti-oxidant effects on cardiomyocytes under pro-inflammatory conditions (LPS) and under exposure to doxorubicin (DOXO). **A** Intracellular reactive oxygen species (iROS) quantification (expressed as cell fluorescence); (n = 3) **B** Quantification of lipid peroxidation products (4-HNA and MDA) (expressed in nmol/mL) (n = 3); **C** Production of nitric oxide (µM) (n = 3). Experiments were performed in cardiomyocytes (5 × 10^3^ cells/well seeded in a 24-well plate) untreated (control) or treated with gallic acid (as positive antioxidant control), EMPA (10, 50, 500 nM), LPS (40 ng/ml) or DOXO (100 nM) alone or co-incubated with EMPA at 10, 50 and 500 nM. One-way ANOVA. Values are expressed ± SD. *** P < 0.001; **P < 0.01; * P < 0.05; ns: not significant

Exposure to LPS or DOXO alone increased the iROS content of 2.1- and 2.6-fold, respectively, compared to untreated cells (Fig. 2A). Levels of iROS were 12%, 33.8% and 62.5% lower in cells treated with EMPA/DOXO than DOXO alone (P < 0.001 for all). Notably, the anti-oxidant effects of EMPA at 50 nM were comparable to those of gallic acid, which is a common bioactive anti-oxidant compound (Fig. 2A). EMPA significantly decreased the production of the lipid peroxidation markers MDA and 4-HNA in cardiomyocytes under pro-inflammatory conditions (Fig. 2B). Lipid peroxidation was two-fold higher in cardiomyocytes treated with DOXO than in untreated cardiomyocytes (p < 0.001). DOXO and EMPA at 10, 50 and 500 nM decreased the production of MDA and 4-HNA by 14% and 11% (p < 0.001 for both), 24% and 26% (p < 0.001 for both), 49% and 35% (p < 0.001 for both), respectively, compared to cells treated with DOXO alone (Fig. 2B). Combination treatment of EMPA/DOXO reduced significantly the nitric oxide production (involved in pathogenesis of cardiovascular diseases [35, 36] compared to cells exposed to DOXO (Fig. 2C).

### Quantification of cytokines

**Fig. 3:**
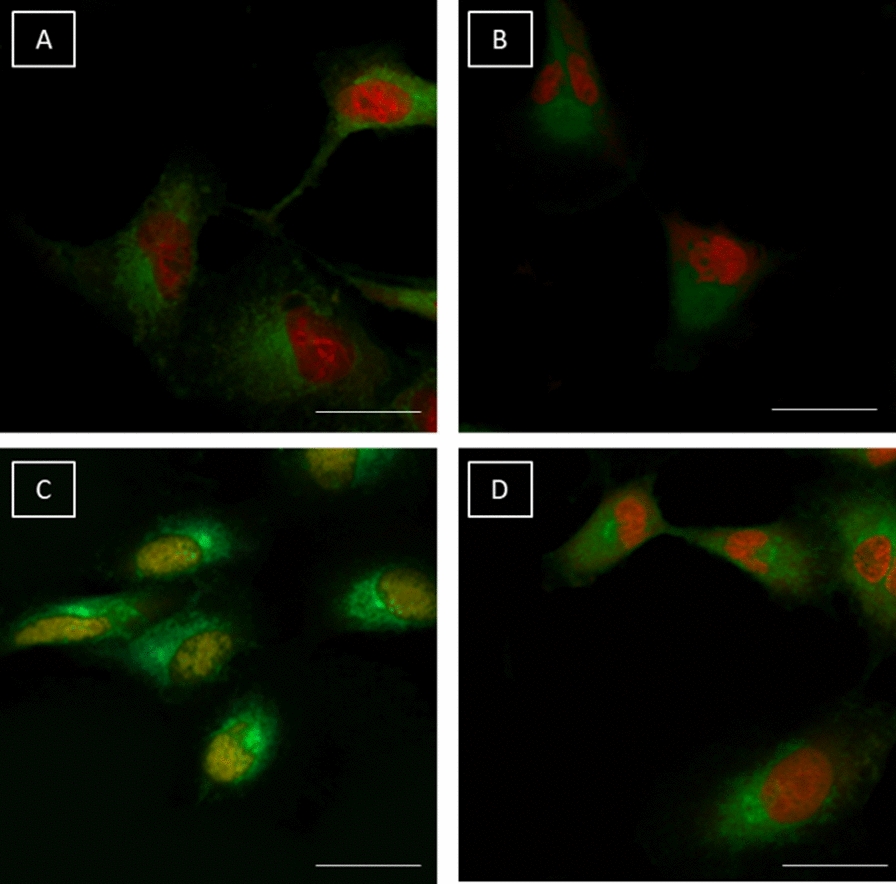
Co-incubation of EMPA (EMPA) and doxorubicin (DOXO) reduced the expression of pro-inflammatory interleukins, leukotrienes and p65/NF-Kb compared to only DOXO-treated cells. For each experiment, 5 × 10^3^ cells/well were seeded in a 96-well plate; cells were exposed to EMPA (10, 50, 500 nM), LPS (40 ng/ml), DOXO (100 nM) alone or with EMPA at 10, 50 and 500 nM (n = 4/group). Expression of (**A**) Interleukin-8, (**B**) Interleukin-6 and (**C**) Interleukin1-β (pg/mL). **D** Leukotriene B4 expression (pg/mL) in cardiomyocytes exposed to arachidonic acid (10 µM), EMPA (10, 50, 500 nM) alone or associated to arachidonic acid, DOXO (100 nM) alone or co-incubated with EMPA at 10, 50 and 500 nM. **E** p65/NF-kB DNA binding activity, expressed as fold of untreated (control) cells (n = 3). (F) MyD88 and NLRP3 expression (fold of control), (n = 3); One-way ANOVA. Value are expressed ± SD. ***P < 0.001; **P < 0.01; *P < 0.05; ns: not significant. **G**: Confocal laser scanning microscope images of cardiomyocytes exposed to medium alone (**A**), EMPA 100 nM (**B**), DOXO 100 nM (**C**) and EMPA (100 nM) DOXO (100 nM) in combination (**D**) for 24 h (n = 3/group). Green fluorescence indicates p65/NF-kB staining; red fluorescence indicates cell nucleus. Scale bar: 50 µM

Interleukin-8 was 2.5-fold higher in cardiomyocytes exposed to DOXO than in untreated cells (Fig. 3A). Treatment with EMPA at 10, 50 and 500 nM resulted in a decrease of interleukin 8 of 8.8% (p < 0.05), 24% (p < 0.001) and 43% (p < 0.001) versus DOXO alone. Interleukin-6, one of the most studied cytokine involved in cardiovascular diseases and cancer progression [37, 38], decreased significantly in cells exposed to EMPA/DOXO (12.4%, 21% and 49.5%, at 10, 50 and 500 nM, respectively, p < 0.001 for all), compared to cardiomyocytes exposed to DOXO alone (Fig. 3B). Moreover, as shown in Fig. 6C, EMPA at 10, 50 and 500 nM reduced the production of interleukin 1-β by 13%, 26% and 48%, respectively (p < 0.001) compared to cells treated with DOXO alone (Fig. 3C).

### Leukotriene B4 assay

Leukotrienes B4 production was 3.4 times higher in cells treated with arachidonic acid than in untreated cells (125.3 ± 5.7 pg/ml vs 36.4 ± 6.7 pg/ml; p < 0.001) (Fig. 3, D). Pre-treatment with EMPA at 10, 50 and 500 nM reduced the production of leukotrienes by 7% (not significant), 16% (p < 0.05) and 33% (p < 0.001) versus cells exposed to arachidonic acid alone (Fig. 3D). Leukotrienes B4 production was 4.3 times higher in cells treated with DOXO than in untreated cells (p < 0.001). Notably, leukotriene B4 production was 24%, 33% and 50% lower in cardiomyocytes treated with DOXO and EMPA at 10, 50 and 500 nM, respectively than in cells exposed to DOXO alone.

### p65/NF-kB assay

EMPA decreased the NF-κB activation in a dose-dependent manner in cardiomyocytes compared to untreated cells (Fig. 3E). Treatment with EMPA at 10, 50 and 500 nM decreased NF-κB activation by 16% (p < 0.05), 32% (p < 0.05) and 53% (p < 0.001) versus cells exposed to DOXO. The same effects of EMPA were recently described by Andreadou I et al. [39].

### MyD88 and NLRP3 expression

The MyD88 complex, also called myddosome and NLRP3 are analysed in cardiac cells exposed to DOXO and EMPA; as shown in Fig. 3F, DOXO strongly increased both pro-inflammatory markers (p < 0.01 for both), in agree with other preclinical studies. Treatment with EMPA decreases significantly MyD88 and NLRP3 indicating their involvement in cardiovascular beneficial effects of SGLT2 inhibitor during DOXO therapy.

### Confocal scanning laser microscope imaging

Cellular imaging confirms the anti-inflammatory effects of EMPA. Cardiomyocytes exposed to DOXO increased significantly the p65/NF-kB expression (Fig. 3C) and nuclear localization compared to untreated cells (Fig. 3A); notably, exposure to EMPA only (Fig. 3B) and combined to DOXO (Fig. 3D) decreased significantly the staining of p65/NF-kB in both in nuclear and cytosol of the cells, indicating anti-inflammatory effects.

## Preclinical studies

### Glycemia

Before treatments, the mean blood glucose was 196.4 ± 43 mg/dl. After treatments with EMPA, the mean blood glucose was 204.6 ± 56 mg/dl and no significant difference were seen between the groups at the end of treatment (p > 0.05). These results are in line with other studies [32] confirming the non-relevant effects of EMPA in non-diabetic mice.

### Effects on ferroptosis, xanthine oxidase and pro-inflammatory biomarkers

**Fig. 4:**
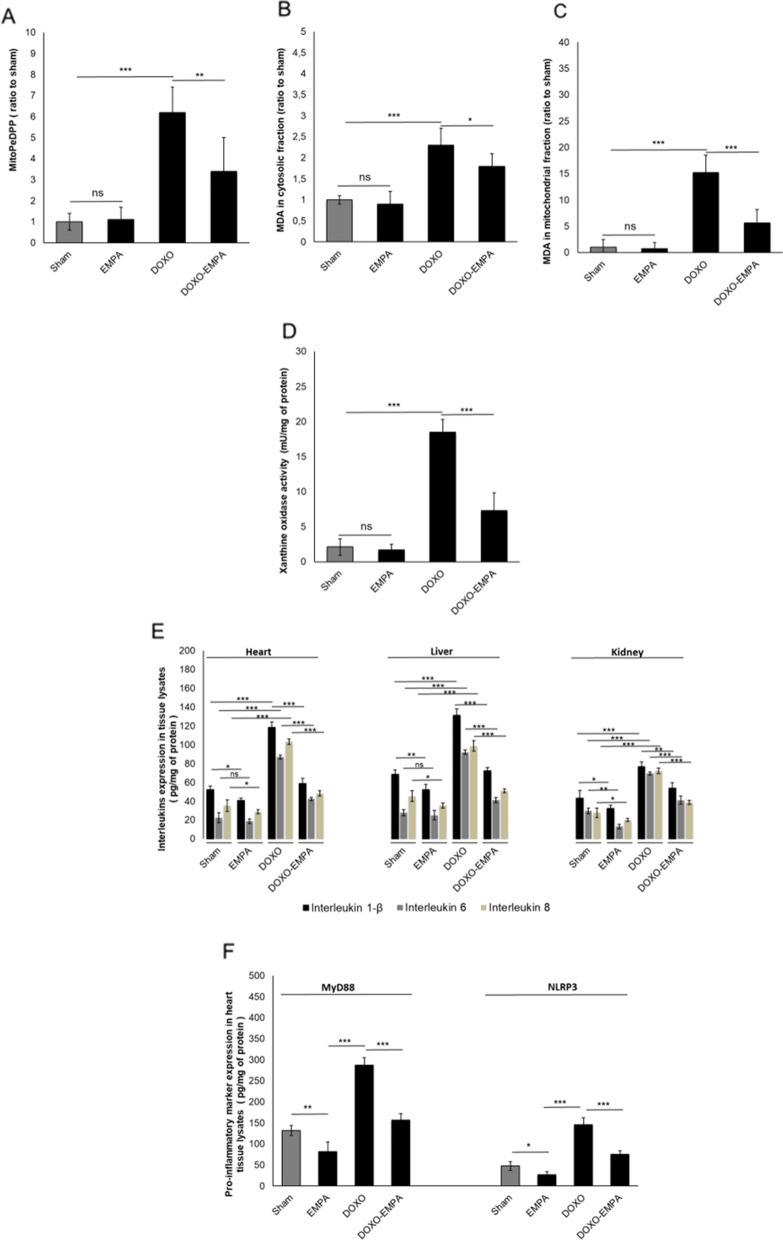
**A** EMPA reduced significantly the DOXO -induced ferroptosis in cardiac tissues, determined by Mitochondrial lipid peroxidation, measured using MitoPeDPP (ratio to sham). EMPA reduced significantly the DOXO -induced MDA content in cytosolic (**B**) and mitochondrial fraction (**C**) of cardiac tissues (ratio to sham). **D** EMPA reduced significantly the xanthine oxidase content in cardiac tissues during treatment with DOXO (mU/mg of protein). **E** EMPA exerts anti-inflammatory effects on the liver, heart and kidney of mice treated with DOXO. **F** EMPA reduces pro-inflammatory markers MyD88 and NLRP3 in cardiac tissue during DOXO treatments. For both, we quantified the expression of interleukin 1-β, interleukin 6 and interleukin 8 (pg/mg of protein) in heart, liver and left kidney lysates of mice untreated (Sham) or treated with EMPA, DOXO or DOXO/EMPA for 7 days (n = 6 for each group); in heart tissues only, we quantified the expression of MyD88 and NLRP3 through mouse ELISA kits (pg of marker/mg of protein). One-way ANOVA. Values are expressed ± SD.*** P < 0.001; **P < 0.01; *P < 0.05; ns: not significant

Increasing evidence indicates that ferroptosis plays a critical role in doxorubicin-induced cardiotoxicity; in agree with literature, DOXO increased MitoPeDPP, a marker of mitochondrial lipid peroxidation involved in ferroptosis (Fig. 4A) and MDA content in cytosolic (Fig. 4B) and mitochondrial (Fig. 4C) fractions of cardiac tissues. Treatment with EMPA during exposure to DOXO reduced significantly ferroptosis and MDA content in the heart, especially in the cardiac mitochondrial fraction (5.6 ± 2.6 vs 15.2 ± 3.3 for DOXO group; p < 0.001). The recent study by Tanaka et al. [31] showed that the increased oxidative stress induced by tissue xanthine oxidase activation is deeply involved in doxorubicin-induced cardiotoxicity; as expected, DOXO increased the expression of xanthine oxidase compared to control (18.5 ± 1.8 vs 2.1 ± 1.2 mU/mg of protein; p < 0.001) (Fig. 4D); in EMPA-DOXO group (Fig. 4E*)* xanthine oxidase is significantly reduced (7.3 ± 2.5 vs 18.5 ± 1.8 mU/mg of protein; p < 0.001). As expected, IL-8, 6 and 1-β expression was significantly higher in the heart, liver and kidney of mice treated with DOXO compared to untreated mice (p < 0.005 for all) (Fig. 4E). Cytokines were significantly lower in EMPA-treated mice than in DOXO-treated mice (p < 0.001 for all)*.* Specifically, IL-1β, IL-6 and IL-8 cardiac expression were 51%, 52% and 54.2% lower, respectively, in EMPA-treated mice than in DOXO-treated mice. In liver, the expression of IL-1β, IL-6 and IL-8 was 45%, 56% and 48%, respectively lower in mice treated with DOXO alone than in mice treated with DOXO plus EMPA. Similar findings were obtained in kidney tissue extract, i.e., IL-1β, IL-6 and IL-8 expression was 29.5%, 41% and 47% lower in mice treated with DOXO plus EMPA than in mice treated with DOXO alone (Fig. 4E). Considering that the NLRP3 and MyD88 are key activators of cytokine storm involved in heart failure, we evaluated their expression in cardiac tissue (Fig. 4F*)*. MyD88 and NLRP3 expression (pg/mg of protein) were significantly reduced by 35–40% (p < 0.001) compared to DOXO group, indicating anti-inflammatory effects (Fig. 4F).

### Effects on fibrosis and apoptosis and SGLT2 expression

**Fig. 5:**
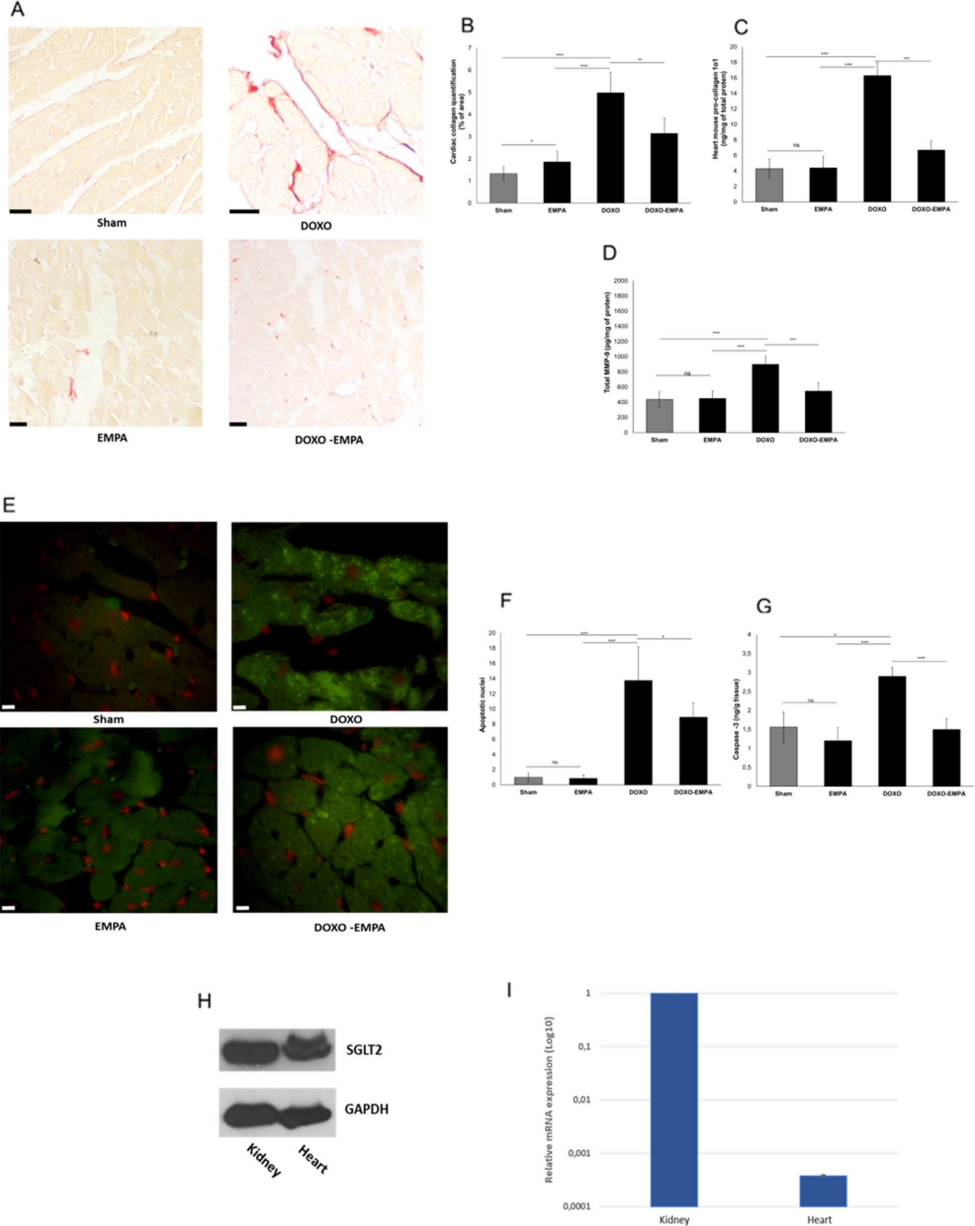
Cardiac fibrosis and apoptosis in mice treated with saline solution (Sham), EMPA 10 mg/kg/day, DOXO 2.17 mg/kg/day or EMPA associated to DOXO (n = 6 for each group). **A** representative images of the interstitial fibrosis (collagen content, indicative of the fibrosis) in cardiac tissue. **B** cardiac collagen quantification (% of collagen of area) in heart tissue. **C** Heart pro-collagen 1-α1 quantification in cardiac tissue (ng/mg of total protein); **D** MMP-9 content ( pg/mg of protein) in cardiac tissue, indicative of fibrosis. **E** apoptosis imaging in cardiac tissue ( in green, apoptotic nuclei). **F** apoptotic nuclei, expressed as relative percentage of positive nuclei in heart tissue (two-way ANOVA with a Bonferroni post hoc test). **G** Caspase-3 expression in cardiac tissue (expressed as ng of Caspase-3/g of tissue). **H** SGLT-2 protein expression in cardiac and renal tissues of C57B/6 mice through western blot analysis. **I** Real Time -PCR analysis indicating the relative mRNA expression of SGLT-2 in cardiac and renal tissues of C57B/6 mice. Values are expressed ± SD. *** P < 0.001; **P < 0.01; * P < 0.05; ns: not significant

Expression of cardiac collagen (imaged in red in the histological pictures) (Fig. 5A, B) was significantly higher DOXO group compared to untreated mice. Treatment with EMPA during exposure to DOXO reduced its expression. Quantitative data confirms that pro-collagen 1α1 expression in EMPA-DOXO group was significantly reduced compared to DOXO (6.7 ± 1.2 vs 16.3 ± 1.7 ng/mg of total protein; p < 0.001) (Fig. 5C). MMP-9 expression, another biomarker of cardiac fibrosis and heart failure, was also reduced in EMPA-DOXO group compared to DOXO (547.3 ± 111 vs 899.6 ± 106 pg/mg of total protein; p < 0.001) (Fig. 5D). Apoptotic nuclei in cardiac tissue (imaged as green signals in Fig. 5E and quantified in Fig. 5F) were 15.8-fold more numerous in DOXO-treated than in untreated mice, and the number of apoptotic events was 35% lower in EMPA-treated mice than in DOXO-treated mice(p < 0.001). Caspase-3 expression in cardiac tissues was also reduced in EMPA-DOXO group compared to DOXO (1.5 ± 0.28 vs 2.9 ± 0.24 ng/g of tissue; p < 0.001) (Fig. 5G) indicating anti-apoptotic effects of EMPA in preclinical models. Notably, western blot analysis (Fig. 5H) showed a very high expression of SGLT-2 in kidney and confirm its expression in cardiac tissue, in line with other studies [32]. RT-PCR confirmed detectable levels of the SGLT-2 mRNA in cardiac tissues (Fig. 5I).

### Effects on cardiac functions

**Fig. 6:**
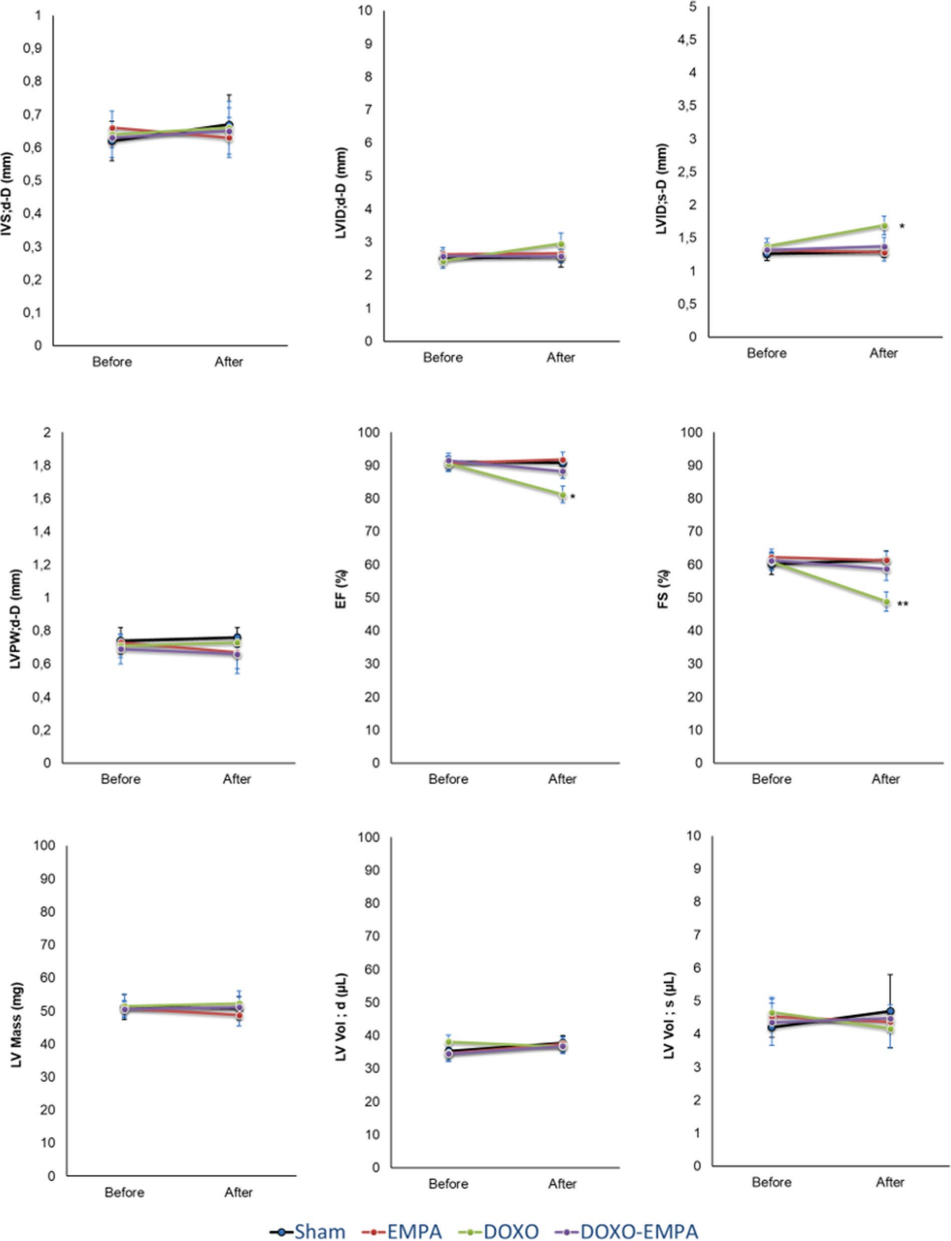
Cardiac function studies in mice treated with saline solution (Sham), EMPA 10 mg/kg/day, DOXO 2.17 mg/kg/day or EMPA plus DOXO (n = 6 for each group). Cardiac function parameters IVS;d-D (mm), LVID;d-D (mm), LVID;s-D (mm), LVPW;d-D (mm), EF (%), FS (%), LV Mass (mg), LV Vol; d (µl), LV Vol; s (µl) were determined through non-invasive transthoracic echocardiography Vevo 2100. (two-way ANOVA with a Bonferroni post hoc test). Values are expressed ± SD. *** P < 0.001; **P < 0.01; * P < 0.05; ns: not significant

No significant differences were observed for IVS;d-D, LVID;d-D, LVPW;d-D, LV Mass, LV Vol; d, LV Vol;s between the experimental groups (Fig. 6). On the other hand, treatment with doxorubicin was associated with a significant increase in LVID; s-D and reduction in EF (− 10.3% compared to baseline: p < 0.05) and FS (− 19.8% compared to baseline; p < 0.05). EMPA-DOXO mice had a significant improvement in EF (88.3 ± 2.3 vs 81.2 ± 2.5 (%); p < 0.05) and FS (58.7 ± 3.5 vs 48.8 ± 2.9 (%); p < 0.05) compared to DOXO group (Fig. 6).

**Fig. 7:**
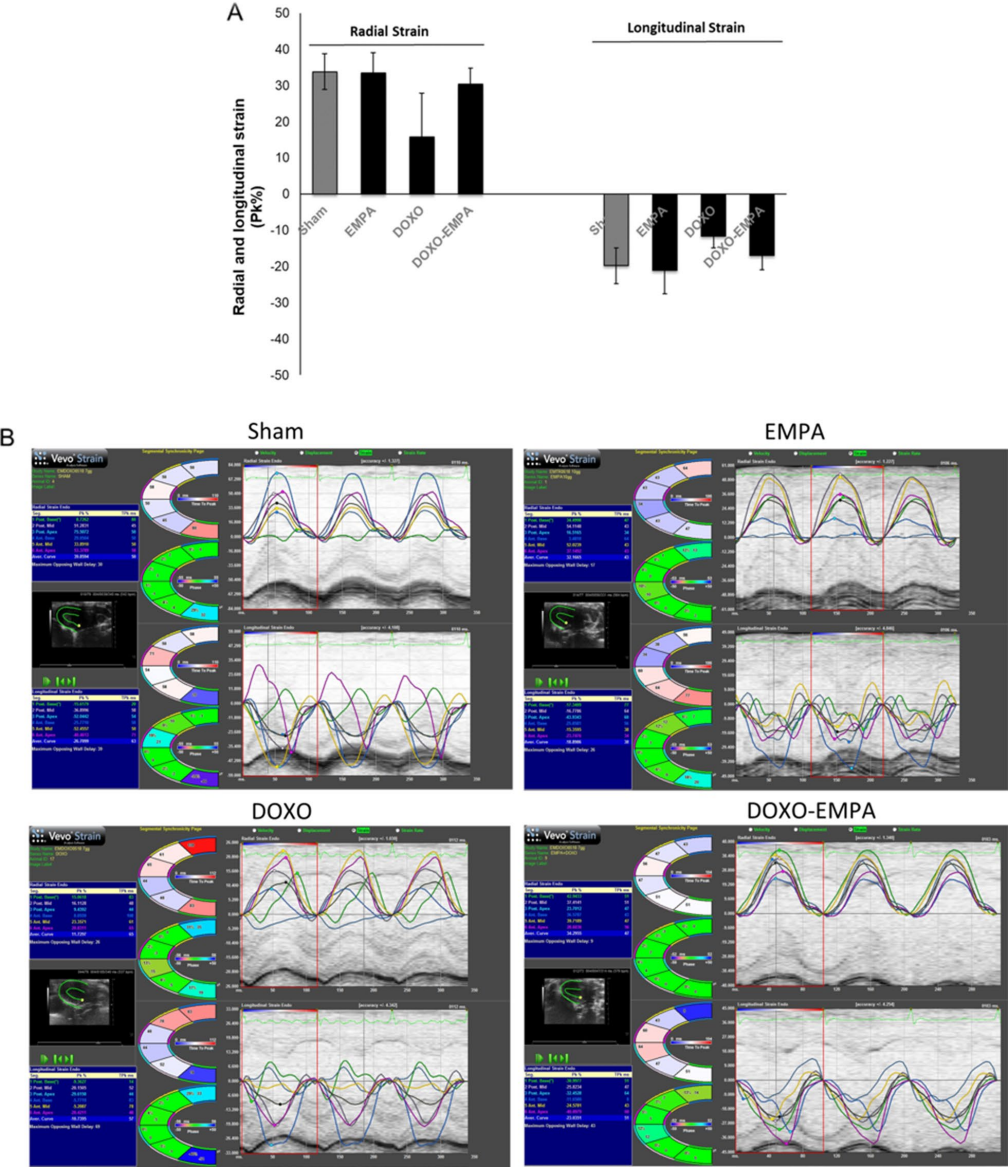
Cardiac function studies in mice treated with saline solution (Sham), EMPA 10 mg/kg/day, DOXO 2.17 mg/kg/day or EMPA plus DOXO (n = 6 for each group). **A** Radial and Longitudinal Strain are expressed as Pk (%). **B** representative radial and longitudinal strain images for each group. (two-way ANOVA with a Bonferroni post hoc test). Values are expressed ± SD. *** P < 0.001; **P < 0.01; * P < 0.05; ns: not significant

Particular attention was made on the evaluation of cardiac strain on long-axis images and ventricular functions (studied by myocardial deformation along the radial and longitudinal axes) as useful echocardiographic markers of cardiotoxicity [40–43]. Strain analysis showed that EMPA significantly improves cardiac functions when used in combination with DOXO compared to DOXO treated mice (quantitative data in Fig. 7A; imaging analysis in Fig. 7B). Radial strain (RS) is 30.3% in EMPA-DOXO vs 15.7% in DOXO groups (P < 0.001); longitudinal strain (LS) is − 17% in EMPA-DOXO vs – 11.7% in DOXO groups (P < 0.001) (Fig. 7A). Moreover, the association of EMPA and DOXO brings strain levels similar to those of untreated mice (control) (Fig. 7A), clearly indicating a cardioprotective effect of EMPA in DOXO-induced cardiotoxicity.

## Discussion

In patients with type 2 diabetes and established cardiovascular disease, EMPA reduced the risk of cardiovascular death and heart failure hospitalizations in the EMPA-REG OUTCOME® trial [8, 9]. More recently, EMPA reduced heart failure hospitalizations and cardiovascular mortality in both high and low risk patients thereby confirming its robust and significant cardioprotective effects [44].

DOXO-induced cardiotoxicity is induced by several mechanisms mediated by increased levels of iROS and [Ca^2+^]i in cardiomyocytes associated to a pro-inflammatory cytokine storm leading to apoptosis, necrosis and mitochondrial dysfunctions. Moreover, treatment with doxorubicin leading to a systemic and local inflammation in cancer patients, partially due to cytosolic damages several organs like liver and heart; doxorubicin exposure in liver increased the production of circulating IL-1-β, IL-6 and hs-CRP (hypersensitive-C-reactive-protein) leading to increased risk of cardiovascular and metabolic diseases [45]. Other preclinical and clinical studies evidenced direct damages induced by doxorubicin, partially mediated by reduced AMPK expression and induction of double-strand breaks and cell death by intercalating into DNA and blocking the activity of the topoisomerase II (TOP2) enzymes called TOP2β and TOP2α [46]. Therefore, consolidated that EMPA exerts systemic and cardiac anti-inflammatory effects in preclinical and clinical trials, its use during treatment with doxorubicin is a promising cardioprotective strategy.

A recent preclinical study suggests that EMPA could reduce intracellular Ca^2+^ under high glucose [47]. It is feasible that EMPA exerts this effect by inhibiting the Na^+^/H^+^ exchanger, thereby leading to a lower concentration of intracellular Ca^2+^ in cardiomyocytes [48, 49]. Given that DOXO exerts cardiotoxic effects by increasing intracellular Ca^2+^concentration [12, 49] it is feasible that the improvement of calcium homeostasis contributes to the cardioprotective effects of EMPA and cardiomyocyte contractility.

Recent preclinical and clinical studies demonstrated that oxidative stress is one of the most important events implicated in the cardiotoxicity of anticancer drugs [50]. Interestingly, EMPA exerts antioxidant effects in cardiomyocytes thereby reducing lipid peroxidation during incubation with DOXO [51–53]. Notably, NO is over-produced in anthracycline-treated patients [54] thereby increasing the risk of cardiac failure and cardiomyopathy [55].Our finding that EMPA reduces NO production in cells exposed to DOXO lays the preliminary foundation for preclinical studies of NO homeostasis in cancer patients.

**Fig. 8:**
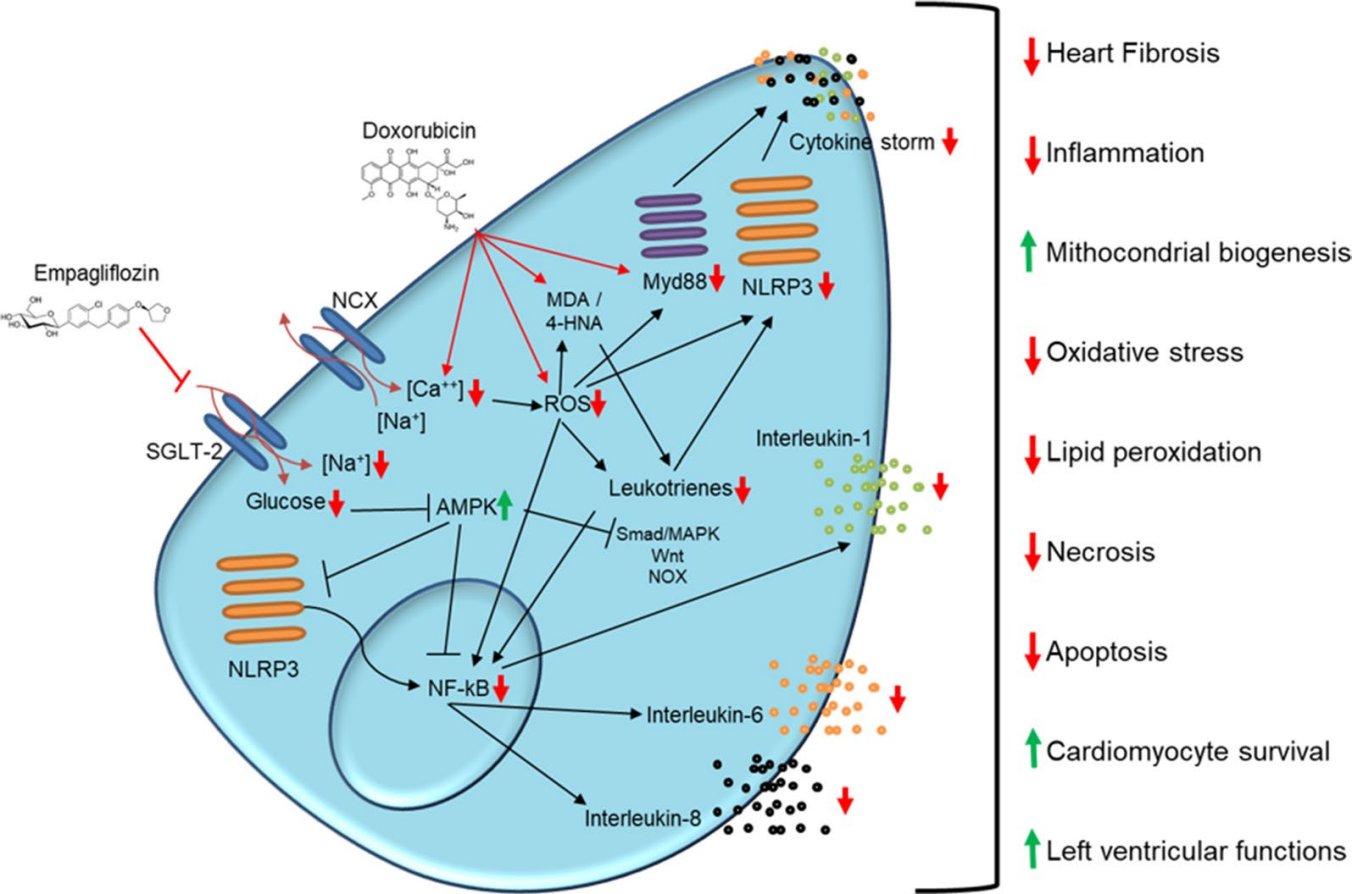
Putative mechanisms of action of EMPA against doxorubicin-induced cardiotoxicity. EMPA inhibits the activity of SGLT-2 thereby reducing intracellular glucose and sodium in cardiomyocytes, resulting in the inhibition of iROS, lipid peroxidation and NLRP3/MyD88-related pathways; the inhibition of NLRP3 and NF-kB reduces the pro-inflammatory cytokine storm in cardiomyocytes exposed to DOXO

The pro-inflammatory heart microenvironment is a key driving force of the cardiotoxicity seen in cancer patients [56–58]. The pharmacological inhibition of IL-1 improves the left ventricle ejection fraction and fraction shortening in preclinical models during DOXO exposure [59]. Interleukins 8 and 6 are also associated to cardiovascular disease, heart failure and stroke [60]. Here, EMPA improved cardiac, hepatic and renal microenvironment through the reduction of pro-inflammatory cytokines during treatment with DOXO. As summarized in Fig. 8, EMPA inhibits activity of SGLT-2 thereby reducing intracellular glucose and sodium in cardiomyocytes, consequently increasing 5' AMP-activated protein kinase (AMPK) that has a key role in doxorubicin-mediated cardiomyocyte injury through SMAD, NoX and Wnt [61]. The overall picture of the study is in line with other recent work highlighting on the protective effects of SGLT2 inhibitors on DOXO induced cardiotoxicity [62, 63]. Interestingly, EMPA exerts cardiorenal benefits that, at least in part, are mediated by the activation of SIRT1 and AMPK signalling pathways acting as adaptive responses cellular stress like chemotherapy [64].

Concentration and administration time of DOXO were in agree with other previous preclinical studies of anthracycline-induced cardiotoxicity [22, 65]; more in details, mice analysed through echocardiography after 10 days of treatment with DOXO to measure left ventricular systolic function, heart rate and cardiac output, were previously described [65, 66] and in accordance to the recommendations of the American Society of Echocardiography [67].

## Study limitations

This study is characterized by some limitations; first, in the clinical scenario, DOXO-induced cardiotoxicity occurs also many years after chemotherapy, especially in young women with breast cancer; however, immediate (acute) cardiac and endothelial cellular damages are frequently diagnosed and are however clinically relevant. Here, we used a preclinical model of acute doxorubicin cardiotoxicity, in agree with previous published works of Tocchetti G et al. [20] where doxorubicin showed significant subclinical damages even after 10 days of treatment (reduced ventricular ejection fraction and acute inflammation). Second, unfortunately, we have not quantified circulating Troponin-T and BNP levels in mice due to unavailability of the kits, however further studies will be performed in order to analyze the effect of EMPA on circulating markers of cardiac damages during treatment with cardiotoxic drugs. Effects on EMPA on the AMPK expression should be investigated both in cellular and preclinical models [68–70]. Other studies will be performed with dapagliflozin or canagliflozin aimed to compare the beneficial effects of different SGLT-2i during treatment with doxorubicin.

## Clinical perspective

Currently, there is a need of cardioprotective strategies in cancer patients treated with doxorubicin, considering its relevant cardiotoxicity [71]. Actually, angiotensin antagonists, statins, beta-blockers and nutraceuticals are under investigations but with no clinically significant beneficial effects. We believe that the systemic anti-inflammatory effects of EMPA and its beneficial properties on cardiac functions in preclinical models treated with doxorubicin may be of useful clinical significance in the primary prevention of heart damages from anthracyclines.

## Conclusion

The present study identified the mechanism whereby EMPA exerts anti-inflammatory and cardioprotective effects in DOXO-induced cardiotoxicity, thereby providing a new therapeutic option for patients undergoing anthracycline-based therapy. The cardioprotective effects of EMPA are biochemically explained by an improvement of the myocardial pro-inflammatory microenvironment and a reduced pro-oxidative state. This study provides the proof of concept for translational studies designed to investigate the cardioprotective use of EMPA during treatments with doxorubicin in cancer patients.

## Data Availability

The data used and/or analysed during the current study are available from the corresponding author on reasonable request.

## Abbreviations

DOXO: Doxorubicin
DOXO + EMPA: Doxorubicin plus EMPA
LV: Left ventricular
EF: Ejection fraction
LS: Longitudinal strain
EMPA: EMPA
SGLT-2i: Selective inhibitor of the sodium glucose co-transporter 2
FS: Fractional shortening
SD: Standard deviations
RS: Radial strain
NHE: Na +/H + exchanger
iROS: Intracellular reactive oxygen species
MDA: Malondialdehyde
4HNA: 4-Hydroxy-nonenoic acid
NLRP3: NOD-, LRR- and pyrin domain-containing protein 3
MyD88: Myddosome type 88
